# Diffraction anisotropy and paired refinement: crystal structure of H33, a protein binder to interleukin 10

**DOI:** 10.1107/S160057672300479X

**Published:** 2023-06-16

**Authors:** Petr Kolenko, Pavel Mikulecký, Phuong Ngoc Pham, Martin Malý, Bohdan Schneider

**Affiliations:** a Czech Technical University in Prague, Brehova 7, Prague 115 19, Czech Republic; b Institute of Biotechnology of the Czech Academy of Sciences, Biocev, Průmyslová 595, Vestec 25250, Czech Republic; Universität Hamburg, Germany

**Keywords:** anisotropy, paired refinement, binder H33

## Abstract

Binder H33 is a small protein binder engineered by ribosome display to bind human interleukin 10. Crystals of binder H33 display severe diffraction anisotropy. A set of data files with correction for diffraction anisotropy based on different local signal-to-noise ratios was prepared. Paired refinement was used to find the optimal anisotropic high-resolution diffraction limit of the data: 3.13–2.47 Å. The structure of binder H33 belongs to the 2% of crystal structures with the highest solvent content in the Protein Data Bank.

## Abstract 2

The paired refinement procedure was applied on diffraction data from binder H33 corrected for strong diffraction anisotropy.

## 1. Introduction

The diffraction quality of a crystal is usually different in various reciprocal space directions. Diffraction anisotropy can be caused by crystal growth, the crystal shape, the modulated volume of the irradiated crystal during the measurement and the arrangement of molecules inside the crystal. This phenomenon is often not a serious issue for a successful structure determination. Most of the macromolecular refinement programs are able to work with weak diffraction anisotropy. But severe diffraction anisotropy may represent a serious threat. The difficulties may appear in the process of phasing and/or structure refinement. However, several computational tools have been developed to analyse or even account for diffraction anisotropy, *e.g. AIMLESS* (Evans & Murshudov, 2013 ▸), *STARANISO* (Tickle *et al.*, 2018 ▸) and *Diffraction Anisotropy Server* (Strong *et al.*, 2006 ▸). These methods perform anisotropic cut-off of the data together with rescaling of intensities or structure factors with scales depending on the analysis and model of anisotropy employed by each program. These modifications are beneficial for a large number of crystal structures and are reported in Section 2 (Rupp, 2018 ▸).

Paired refinement is a modern method for determining the high-resolution cut-off of diffraction data (Karplus & Diederichs, 2012 ▸). For this method, the reference data are selected on the basis of a conservative cut-off [*e.g.* 〈*I*/σ(*I*)〉 < 2]. More and more reflections are added to the model refinement in a stepwise manner, and their positive or negative contribution is evaluated on a number of criteria, mainly *R*_free_ calculated on the reference data. Recently, the method has been implemented in the program *PAIREF* (Malý *et al.*, 2020 ▸, 2021 ▸). However, the current protocol implemented in *PAIREF* does not consider the anisotropic diffraction qualities of the crystals. Both the reference data and the evaluated reflections are in the form of spherical shells.

We investigated the possibility of combining corrections for diffraction anisotropy with the standard paired refinement approach. The crystals of our target protein H33 showed serious anisotropy in the diffraction qualities. H33 is an artificial protein binder that was selected during a directed evolutionary study (Pham *et al.*, 2021 ▸); it is a variant of the protein scaffold derived from the N-terminal domain of the PIH1D1 domain of the R2TP cochaperone complex (PDB entry 4psf; Hořejší *et al.*, 2014 ▸). The scaffold was trained using the ribosome display technique to bind human interleukin-10 (IL-10), a cytokine of human innate immunity (El Kasmi *et al.*, 2007 ▸). Blocking or potentiating IL-10 signalization by artificially evolved non-antibody binders such as H33 could be an important component of the treatment of inflammatory, malignant and autoimmune diseases in which IL-10 plays a role. However, our understanding of the structural aspects of the binding between the binders and IL-10 is quite limited. So far, we have solved the structure of only one IL-10 binder called J61 (PDB entry 7avc; Pham *et al.*, 2021 ▸). Therefore, a newly solved structure of H33 will aid in the design of new potent and selective binders.

In our work, we introduced an approach to perform paired refinement using the anisotropic data. Anisotropic scaling proved to have a positive impact on the quality of the observed electron density.

## 2. Materials and methods

### 2.1. Protein production and crystallization

Protein production, purification and basic characterization have been described previously (Pham *et al.*, 2021 ▸). Briefly, the synthesized DNA strings were cloned into the pET-26*b*(+) vector. The plasmid was used for transformation into the *Escherichia coli* strain BL21(DE3). The bacteria were grown in LB medium, and protein expression was induced by the addition of iso­propyl-beta-d-thiogalactopyran­oside. After cell disruption, the soluble fraction was separated by centrifugation and the protein was purified from the cell lysate by affinity chromatography using Strep-Tactin XT resin. The last purification step was performed using size-exclusion chromatography (Superdex 75 16/600 column).

The crystals were prepared using the hanging-drop vapour-diffusion method from a protein solution that contained 20 m*M* Tris, 100 m*M* NaCl pH 8.0 and the protein at a concentration of 10 mg ml^−1^. The protein crystallized in a wide range of crystallization conditions. However, the crystals diffracted poorly. The final crystallization conditions were 1 *M* (NH_4_)_2_SO_4_, 1%(*w*/*v*) PEG 3350, 0.1 *M* bis-Tris pH 5.5. Cryoprotection with 20%(*v*/*v*) glycerol was necessary before flash-freezing in liquid nitro­gen.

### 2.2. Diffraction data collection and processing

The synchrotron data were collected on beamline P13 (Cianci *et al.*, 2017 ▸) operated by EMBL Hamburg at the PETRA III storage ring (DESY, Hamburg, Germany). The diffraction images were processed with *XDS* (Kabsch, 2010 ▸) up to 2.3 Å resolution. The data quality metrics [decrease in 〈*I*/σ(*I*)〉, decrease in *CC*_1/2_] indicate radiation damage that started immediately after 180° of total oscillation and progressed to the end of the measurement. Therefore, only half of the images (3600) were used for further data evaluation. Such data treatment should remove the possible impact of absorbed dose on the resulting diffraction anisotropy. Initial scaling of the data was performed using *AIMLESS* (Evans & Murshudov, 2013 ▸). The data were severely anisotropic according to a number of indicators. For example, the estimates of the diffraction limits reported by *AIMLESS* [based on criterion for 〈*I*/σ(*I*)〉 > 1.5 in the highest-resolution shell] were 3.28 and 2.65 Å along the *hk* plane and the *l* axis, respectively.

Due to severe diffraction anisotropy, the unmerged scaled data from *XDS* (XDS_ASCII.HKL file) were merged and corrected for anisotropy using the *STARANISO* server (Tickle *et al.*, 2018 ▸) with four different local spherical 〈*I*_mean_/σ(*I*_mean_)〉 cut-offs going down from 1.2 (*STARANISO* default value, A1.2 data) to 1.0 (A1.0 data), 0.75 (A0.75 data) and the lowest available value 0.5 (A0.5 data). The free flags were generated with the program *FREERFLAG* (Brünger, 1992 ▸). Initially, free flags were generated for the A1.2 data. The free flags for the A1.0 data were generated with the option to copy already existing flags for reflections in the A1.2 data. A similar approach was used for the generation of free flags for the A0.75 and A0.5 data. This approach was necessary to maintain the pairwise consistency of the free flags within the different data. Data quality indicators are shown in Table 1 ▸.

The phase problem was solved by molecular replacement using *PHASER* (McCoy *et al.*, 2007 ▸) employing the J61 variant of the protein binder from the same directed evolutionary study (PDB entry 7avc; Pham *et al.*, 2021 ▸) as a search model. Data with the highest 〈*I*_mean_/σ(*I*_mean_)〉 cutoff (A1.2) were used. Two molecules were found in the asymmetric unit. Due to the low resolution of the data and the unstable refinement (unacceptable number of Ramachandran outliers and bad bond angles) in *REFMAC5* (Murshudov *et al.*, 2011 ▸), the structure was restrained to the original scaffold of the PDB entry 4psf refined at 1.58 Å (Hořejší *et al.*, 2014 ▸) with *PROSMART* (Nicholls *et al.*, 2012 ▸). The structure was refined with isotropic atomic displacement parameters (ADPs) and no TLS domains defined. Manual corrections to the model were performed with *Coot* (Emsley *et al.*, 2010 ▸).

For the manually launched paired refinement, the data with the highest cut-off (A1.2) were initially chosen. Refinement of the structure model restrained to the structure of PDB entry 4psf with *REFMAC5* was used. To keep the same refinement scheme as used previously, three cycles were performed in each paired refinement step. We also performed several manual paired refinements with ten cycles of refinement. Although the results differ in exact values, this change did not lead to a different decision on data usage. Several criteria were evaluated during the paired refinement. Mainly, drops in overall *R*_work_ and *R*_free_ were monitored. In addition to that, *R*_free_ in the highest-resolution shell did not exceed the value of 0.42 (the theoretically perfect model gives an *R* value of 0.42 against random data with no twinning and no translational non-crystallographic symmetry; Evans & Murshudov, 2013 ▸), and the values of *CC*_work_ and *CC*_free_ did not exceed the value of CC* (see Table 1 ▸). The main results of the paired refinement are shown in Table 2 ▸. The decrease in *R*_work_ and *R*_free_ values in all three steps indicates that the addition of the progressively weaker reflections improved the model quality against the same (stronger) data. Therefore, A0.5 data were used in further structure refinements. The exact values of the final *R*_work_ and *R*_free_ in the fifth column of Table 2 ▸ cannot be directly compared because they were calculated against different data. Although the differences in the *R*_work_ and *R*_free_ values can be considered marginal, they are comparable to values published in previous studies (Karplus & Diederichs, 2012 ▸; Malý *et al.*, 2020 ▸, 2021 ▸).

The final model refinement using ten cycles was carried out using all reflections (work and free) of the A0.5 dataset. Jelly body protocol was used to release the previously used and necessary restraints. The quality of the structure stereochemistry was checked using the validation tools in *Coot* (Emsley *et al.*, 2010 ▸), *CCP4* (Agirre *et al.*, 2023 ▸; Winn *et al.*, 2011 ▸), *MOLPROBITY* (Chen *et al.*, 2010 ▸) and the Protein Data Bank (Berman *et al.*, 2003 ▸). The quality indicators of the final structure refinement are shown in Table 3 ▸. Raw diffraction data are available from https://doi.org/10.5281/zenodo.4033811. The structure coordinates were deposited under PDB entry 8bdu.

For analysis of the additional value of anisotropic scaling along with paired refinement in terms of data quality and observed electron density, data processed in the standard (isotropic) way were used in paired refinement with a 2.9 Å starting resolution. The complete cross-validation procedure implemented in *PAIREF* extended the resolution to 2.8 Å.

## 3. Results and discussion

The artificially generated binder H33 was successfully crystallized and the diffraction data were collected. The crystal diffracted anisotropically, and correction of the intensities for diffraction anisotropy was performed. The crystal structure was solved and refined using the A1.2 data. The high-resolution diffraction limit was extended using the paired refinement procedure to that of the A0.5 data. The A0.5 data were used for final structure refinement.

The structure of binder H33 is highly similar to that of binder J61 (Pham *et al.*, 2021 ▸) from the same study. The root mean square deviation calculated on 128 Cα atoms is lower than 1.2 Å. The structure has an unusually high solvent content of 76% (Kantardjieff & Rupp, 2003 ▸). This high solvent content is present in <2% of the crystal structures in the PDB. The solvent content is probably responsible for the low diffraction quality of the crystals.

**Figure 1:**
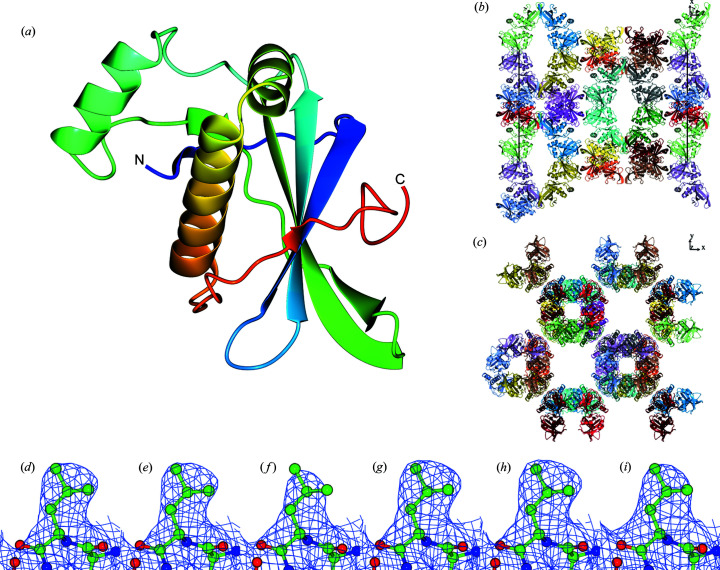
(*a*) Structure of the monomeric binder H33 determined using the A0.5 data in a secondary structure representation. (*b*)–(*c*) Unit cell filled with molecules viewed from different perspectives. (*d*)–(*f*) Residue Leu75 (chain A) with the 2m*F*_o_ − D*F*_c_ electron density (blue mesh) at the level of 0.15 e Å^−3^ calculated for the model before the paired refinement procedure using data A1.2, the output model from the paired refinement procedure using the A0.5 data and the model from the isotropic paired refinement procedure using the Iso data, respectively. (*g*)–(*i*) Residue Leu75 (chain A) with the 2m*F*_o_ − D*F*_c_ electron density at the 1σ level for the same combination of model versus data as in the previous triplicate. Graphics were generated with *CCP4MG* (McNicholas *et al.*, 2011 ▸).

Previous studies have shown that diffraction anisotropy is not strictly dependent on crystal packing (Robert *et al.*, 2017 ▸). The molecules in the crystal of binder H33 are arranged in tubules perpendicular to the *z* axis of the crystal lattice. The tubules have large channels of solvent between them. The planes with the normal vector perpendicular to the *z* axis are the least occupied with molecules [see Fig. 1 ▸(*b*)]. In contrast, no large channels are present in the planes with normal vectors perpendicular to the *x* or *y* axis.

Using the data range according to paired refinement may result in an improvement of the observed electron density (Karplus & Diederichs, 2015 ▸; Malý *et al.*, 2020 ▸). Correction for the anisotropy in the diffraction qualities was also shown to improve the observed electron density (Tickle *et al.*, 2018 ▸). Paired refinement using shells reflecting the diffraction anisotropy is not automated in any pipeline. The available software, for example *PAIREF* (Malý *et al.*, 2020 ▸) and *PDB-REDO* (Joosten *et al.*, 2014 ▸), use the addition of reflections in spherical shells by increasing the spherical high-resolution diffraction limit. Here, we propose the addition of reflections with the same expected information content in non-spherical shells.

The quality of the electron density depends on the diffraction data and the structural model. In our analysis, we compared electron density maps of (i) the starting model for the paired refinement refined using the A1.2 data against the A1.2 data, (ii) the resulting model from the paired refinement refined using the A0.5 data against the A0.5 data and (iii) the optimal model from the paired refinement using the Iso data at 2.8 Å resolution. The electron density maps were calculated with fast Fourier transformation using the same grid spacing to avoid possible bias (Urzhumtsev *et al.*, 2014 ▸). The electron density map from the Iso data is the least detailed [see Fig. 1 ▸(*f*)]. Corrections for diffraction anisotropy using the *STARANISO* server (Tickle *et al.*, 2018 ▸) and using the A1.2 data dramatically improved the quality of the observed electron density maps. No differences were observed with the extension of data from A1.2 to A0.5.

The number of reflections in the A0.5 data is approximately equal to that of the Iso data. Apparently, their information content is different. The anisotropic cut-off in the A0.5 data removed a significant portion of noisy reflections. The Iso dataset contains reflections in the weak directions with a low signal-to-noise ratio. Moreover, it does not contain a portion of the strong reflections in the strong directions that are present in the A0.5 data at a resolution higher than 2.8 Å.

In our case, both approaches to data optimization (correction for diffraction anisotropy and paired refinement) have proved useful. Although improvement in observed electron density did not occur after paired refinement of data corrected for diffraction anisotropy, 2676 unique reflections (14.5% from 18 374 reflections in total) were added to the refinement scheme using the A0.5 data. This addition was validated by the decrease in *R* values (see Table 2 ▸).

The current trend in data quality evaluation (paired refinement) is to investigate the ‘additional value’ of more and more observations involved in model refinement. Conventional indicators of the quality of the diffraction data are no longer relevant for the estimation of the high-resolution diffraction limit. The diffraction anisotropy makes the problem even more difficult. Our crystal structure was determined at a nominal diffraction limit of 2.47 Å. However, closer inspection of the diffraction data statistics shows that the diffraction data become dramatically incomplete at better than 3.13 Å resolution. The highest-resolution shell of reflections has a spherical completeness lower than 15%. This indicator must be considered when comparing structures refined ‘with the same diffraction limits’.

## Supplementary Material

PDB reference: 8bdu

URL: https://doi.org/10.5281/zenodo.4033811

## Figures and Tables

**Table 1:** Data collection and processing statistics Values in parentheses are for the highest-resolution isotropic shell.

Diffraction source	Petra III, P13				
Wavelength (Å)	0.976				
Temperature (K)	100				
Crystal-to-detector distance (mm)	576.1				
Exposure per image (s)	0.04				
Images collected/processed	7200/3600				
Space group	*I*4_1_22				
*a* = *b*, *c* (Å)	123.13, 190.11				

Dataset	A1.2	A1.0	A0.75	A0.5	Iso
Local 〈*I*/σ(*I*)〉 cut-off	1.2	1.0	0.75	0.5	Isotropic data
Resolution range (Å)	47.53–3.29†–2.58 (2.83–2.58)	47.53–3.26†–2.56 (2.80–2.56)	47.53–3.20†–2.52 (2.76–2.52)	47.53–3.13†–2.47 (2.71–2.47)	47.53–2.80 (2.95–2.80)
Total No. of reflections	198331 (8000)	204983 (8055)	215735 (8090)	230863 (8194)	237010 (36042)
No. of unique reflections	15698 (785)	16246 (812)	17151 (858)	18374 (919)	18372 (2625)
Spherical completeness (%)	67.4 (14.4)	68.1 (14.6)	68.2 (14.3)	69.2 (14.8)	100 (100)
Ellipsoidal completeness (%)	94.5 (79.7)	94.6 (77.9)	94.3 (73.8)	94.2 (71.3)	N/A
Multiplicity	12.6 (10.2)	12.6 (9.9)	12.6 (9.4)	12.6 (8.9)	12.9 (13.7)
〈*I*/σ(*I*)〉	17.3 (1.9)	16.7 (1.6)	15.9 (1.3)	14.8 (0.8)	14.0 (1.0)
*R*_meas_	0.114 (1.843)	0.117 (2.045)	0.121 (2.546)	0.127 (3.775)	0.129 (5.545)
*R*_pim_	0.032 (0.559)	0.033 (0.629)	0.034 (0.801)	0.036 (1.216)	0.036 (1.476)
*CC*_1/2_	0.999 (0.590)	0.999 (0.529)	0.999 (0.436)	0.999 (0.224)	0.999 (0.628)
CC*	0.999 (0.861)	0.999 (0.832)	0.999 (0.780)	0.999 (0.605)	0.999 (0.878)

† Lowest high-resolution diffraction limit after anisotropic cut-off.

**Table 2:** Progress of paired refinement using data with a continuously decreasing 〈*I*/σ(*I*)〉 cutoff Initial *R* values in *X*→*Y* steps are calculated using the model refined with data *X* against data *X*. Final *R* values are calculated using the model refined with data *Y* against data *X*.

Dataset	Resolution (Å)	Unique reflections added	Initial *R*_work_/*R*_free_	Final *R*_work_/*R*_free_	Δ*R*_work_/*R*_free_
A1.2	47.53–3.29†–2.58			0.2228/0.2560	
A1.2→A1.0	47.53–3.26†–2.56	548	0.2228/0.2560	0.2202/0.2525	−0.0026/−0.0035
A1.0→A0.75	47.53–3.20†–2.52	905	0.2248/0.2572	0.2214/0.2538	−0.0034/−0.0034
A0.75→A0.5	47.53–3.13†–2.47	1123	0.2287/0.2611	0.2247/0.2583	−0.0040/−0.0028

† Lowest high-resolution diffraction limit after anisotropic cut-off.

**Table 3:** Structure refinement statistics Values in parentheses are for the highest isotropic resolution shell.

Resolution (Å)	47.53–3.13†–2.47 (2.71–2.47)
No. of residues/non-H atoms	266/2071
*R*_work_	0.219 (0.355)
*R*_free_	0.255 (0.368)
*CC*_work_	0.993 (0.492)
*CC*_free_	0.913 (0.590)
No. free reflections	910
*R*_all_	0.223 (0.379)
Average ADP (Å^2^)	82
R.m.s. deviations from ideal	
Bond lengths (Å)	0.010
Angles (°)	1.755
Ramachandran plot[Table-fn tfn3]	
Allowed	261 (99.6%)
Outliers	1 (0.4%)
Solvent content (%)	76

† As calculated by *MOLPROBITY*.

‡ The lowest high-resolution diffraction limit after the anisotropic cut-off.
